# Vulnerability indicators for natural hazards: an innovative selection and weighting approach

**DOI:** 10.1038/s41598-019-50257-2

**Published:** 2019-10-21

**Authors:** Maria Papathoma-Köhle, Matthias Schlögl, Sven Fuchs

**Affiliations:** University of Natural Resources and Life Sciences, Institute for Mountain Risk Engineering, Vienna, 1190 Austria

**Keywords:** Natural hazards, Climate-change adaptation

## Abstract

To prepare for upcoming extreme events, decision makers, scientists and other stakeholders require a thorough understanding of the vulnerability of the built environment to natural hazards. A vulnerability index based on building characteristics (indicators) rather than empirical data may be an alternative approach to a comprehensive physical vulnerability assessment of the building stock. The present paper focuses on the making of such an index for dynamic flooding in mountain areas demonstrating the transferability of vulnerability assessment approaches between hazard types, reducing the amount of required data and offering a tool that can be used in areas were empirical data are not available. We use data from systematically documented torrential events in the European Alps to select and weight the important indicators using an all-relevant feature selection algorithm based on random forests. The permutation-based feature selection reduced the initial number of indicators from 22 to seven, decreasing in this way the amount of required data for assessing physical vulnerability and ensuring that only relevant indicators are considered. The new Physical Vulnerability Index (PVI) may be used in the mountain areas of Europe and beyond where only few empirical data are available supporting decision-making in reducing risk to dynamic flooding.

## Introduction

Mountain areas are sensitive to climate change, experience significant socio-economic dynamics, have insufficient space for settlement and often face challenges related to limited communication networks due to their remoteness. In these areas, where actually 12% of the world’s population live^1^, natural hazards, such as dynamic flooding, are capable of causing significant suffering and disruption to local communities. Dynamic flooding is characterized by high flow rate and high sediment concentration. It occurs in mountain rivers and torrents and includes processes such as fluvial sediment transport, debris flow and related phenomena that repeatedly cause damages to the built environment in mountain areas globally.

Understanding, analysing, quantifying and visualizing vulnerabilities enable authorities, decision makers and other stakeholders managing and reducing existing and emerging risks. Vulnerability is an essential component of risk analysis and, as such, it has to be deeply investigated. The most common approach of assessing physical vulnerability to dynamic flooding is the computation of vulnerability curves^2,3^. However, these curves are associated with a large number of uncertainties deriving from the fact that the constructive and design characteristics of buildings are often not considered and, instead, empirical relationships between hazard magnitude and observed loss heights are used^2^. An alternative approach could involve the development of a vulnerability index for each building based on a number of indicators (building characteristics and properties). Indicator-based methods are often used for the assessment of social (e.g. SoVI^4^) and eco-environmental vulnerability^5–7^. In limited cases and for hazards other than flooding, physical vulnerability of the built environment has already been successfully approached using indicators. Such an approach is the PTVA (Papathoma Tsunami Vulnerability Assessment), originally proposed for assessing the effects of tsunami hazards. The PTVA has been introduced in 2003^8^ and has been further developed and improved since then^9^. In the present paper a modified version of this method will be used to prove its application for dynamic flooding in mountain areas.

Usually, adequate vulnerability approaches are developed for each hazard type. Methods suitable for one hazard type are rarely modified and used for another one limiting possible synergies between research groups from different disciplines. Although the use of indicators is common for more hazard types there are significant differences concerning the selection and scoring of indicators, scale, unit of study (building, building block, administrative area) and weighting method. For this reason, the transfer of methods from one natural process to the other is not common practice. Moreover, due to the different impact that processes have on the elements at risk a whole new set of indicators have to be collected for each hazard type even if the framework remains the same. Differences are evident also in the scale and unit of research used in different studies. For example, studies focusing on environmental vulnerability (e.g.^5–7^) are often conducted in a regional scale and use as unit of research larger areas based on land-cover and land use types, whereas in the case of physical vulnerability to earthquakes (e.g.^10^) and floods (e.g.^11^) the unit may be the building, the building block or the administrative area depending on the aim of the study. In a cross-disciplinary study for flood and earthquake vulnerability approaches, Ruiter *et al*.^12^ suggest that differences between the two disciplines mainly focus on the scale, vulnerability dimension (social, physical etc.), individual focus (e.g. local economic sector vs. social factors) and existence of temporal components. We attempt herein the transfer of a method used for tsunami to dynamic flooding without making significant changes to it based on the similarities of the two processes.

Occasionally natural processes share similar properties. For example, even if tsunami hazards and dynamic flooding have many differences, as far as the impact on buildings is concerned, they do share some common characteristics. Damage in both cases is related to hydrostatic and hydrodynamic forces as well as buoyancy and uplift forces, debris impact, debris damming, and additional gravity loads from retained water and material on floors above the ground level^13^. Nevertheless, there are also many differences including the type of debris (during the tsunami waterborne debris such as large boats or shipping containers may cause major damage or debris damming). Furthermore, tsunamis contain sediment that does not exceed 10% by volume^13^, whereas during dynamic flooding sediment concentration may exceed 50%^14^. Moreover, in the case of tsunamis additional damage may occur due to the “bore impact” (a broken wave that propagates over still water)^15^ which is not the case for dynamic flooding in mountain catchments. Finally, tsunamis affect large and flat coastal areas, whereas flooding in mountain areas often impact small settlements located on steep slopes or within small catchments. For both types of hazards empirically-derived vulnerability curves have been used to show the relationship between magnitude and the observed degree of loss^16,17^. However, a comparison of these empirical curves is impossible due to the outlined differences in hazard characteristics. Therefore, we test transferability of methods for damage assessment by modifying the PTVA that used indicators to assess the vulnerability of buildings exposed to tsunami impact, and approach the issue of vulnerability for dynamic flooding in mountain catchments.

Birkmann^18^ and Fuchs *et al*.^19^ define indicators as variables that are “an operational representation of a characteristic or quality of a system”. According to OECD^20^, the weighting and the aggregation of indicators to indices may have a large impact on the resulting rankings^21^ and, consequently, on decision making^22^. In the literature, there are many available methods both for weighting and aggregating indicators to indices. According to Gan *et al*.^23^ and Fuchs *et al*.^19^ the choice of the weighting and aggregation method is related to the objective of the study and the spatial and temporal scales used. The use of indicators has many advantages compared to traditional empirical loss assessment, including the fact that complex issues are summarized supporting decision making and communication among stakeholders is facilitated^24^.

Indicator-based methods for physical vulnerability assessment are limited, however, the number of studies using this method is growing rapidly. As far as mountain hazards in torrential catchments are concerned, one of the first attempts in this direction was made by Papathoma-Köhle *et al*.^25^. An inventory of exposed buildings and their characteristics relevant to their susceptibility to landslides including debris flow was generated. The building characteristics (indicators) were weighted according to their contribution to the overall building vulnerability using expert judgement. An improved version of the method had been provided by Kappes *et al*.^26^ and applied in France in a multi-hazard environment. It has been recognized that the most significant drawback of the method is that process intensities were not considered in the index calculation. Silva and Pereira^27^ partly closed this gap by combining the resistance of the building with the magnitude of the process (in this case shallow landslides), an approach that was also chosen by Mazzorana *et al*.^28^ and Milanesi *et al*.^29^, including information such as the construction technique and material, the floor and roof structure, and the number of floors.

Thouret *et al*.^30^ as well as Ettinger *et al*.^31^ used indicators to assess the physical vulnerability of the built environment to debris flow in the Andes. Based on the analysis of high-resolution satellite imagery, Thouret *et al*.^30^ presented an index based on indicators such as building type, number of floors, percentage and quality of building openings and roof type for a large dataset. Using the same data together with ground truth observation, Ettinger *et al*.^31^ reported vulnerability indices based on indicators such as number of stories, building footprint, shape of city block and building density as well as distance from channel, distance from bridge and impermeability of the soil. Finally, Thennavan *et al*.^32^ based on the method of Papathoma-Köhle *et al*.^25^ presented physical vulnerability indices for buildings in India.

The construction of indices requires a number of steps such as indicators selection, weighting and aggregation. In general, indicators selection in the literature has been based until now on arbitrary expert judgement. As far as weighting is concerned, according to Beccari^33^, who reviewed 106 studies on index construction for natural hazard risk assessment, most of the studies were based on equal weighting (44 studies), which means that each individual indicator has the same impact on the final result, followed by weighting based on statistical methods (28 studies), weighting based on participatory methods (19 studies) and weighting based on different – and thus more subjective – approaches by the respective authors (13 studies).

In this paper, a new indicator-based approach based on the PTVA method^34^ is presented based only on relevant data. The method is applied in the European Alps for buildings subject to dynamic flooding. The advantage of the method in comparison to already existing studies is the advanced selection and weighting of the indicators and their predictive power. Thus, the method can be used in regions that have never experienced the impact of dynamic flooding so far, and due to its easy applicability and flexibility, the method may also be used by a wide range of professionals such as practitioners, decision makers, or even by homeowners.

The innovative aspects of the presented approach are outlined as follows:

1. An existing approach, PTVA, is adapted for a different hazard type (dynamic flooding) in mountain areas, giving deeper insights into the interaction between building envelope and hazard dynamics.
2. An all-relevant feature selection entails that only relevant indicators are considered in the construction of the new index, thereby significantly reducing the required amount of data.
3. The indicator weighting is based on indicator importance of real-world damage data from recent events.
4. Results of laboratory experiments are used to support the selection of and relevance between indicators.
5. Given enough data, the method is robust since it is based on an ensemble of tree-based models.
6. A Physical Vulnerability Index (PVI) for dynamic flooding is computed which can be used for a variety of processes without distinguishing between different torrential hazards (flash flood, hyper-concentrated flow, fluvial sediment transport, debris flow).
7. The method is easily transferable to other locations and highly flexible since it can be adapted to local conditions.
8. The PVI can be used in areas without historic record of events and in absence of empirical data to quantify the vulnerability of buildings.
9. The PVI can be used from different end-users including homeowners, local authorities, insurance companies and emergency services.

**Figure 1:**
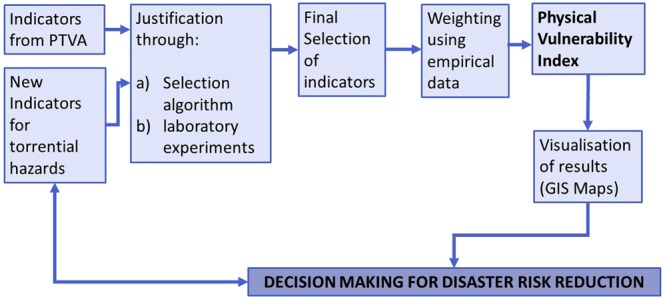
Methodological flow and aim of the study.

The aim of this paper is to describe the adaptation of a method that has originally been established for assessing physical vulnerability for one hazard type to another hazard type occurring in a completely different environment. Specifically, the procedure and making of a new PVI for torrential hazards based on the PTVA method is described (Fig. 1). The paper focuses on the process of indicator selection and weighting based on the importance of building characteristics being tested using real-world damage data. The index constitutes a valuable tool for decision makers since they can have an overview of the vulnerability of buildings at local level. On this basis, they can make decisions regarding evacuation planning, local adaptation measures, larger structural protection measures and necessary reinforcement of buildings. Advantages and limitations of the method as well as possible future improvements are discussed.

The selection and weighting of the indicators index is based on data from four different case studies in the Austrian and the Italian Alps, respectively (Table 1). In most areas the torrential processes were described as fluvial sediment transport and only in a few areas the process was identified as a debris flow. The PVI was individually calculated for all four case studies.

**Table 1:** Characteristics of the 2005 event in the Austrian case study areas^42–45^ and the 1987 event in the Italian case study^46,47^.

Location	Process type	Velocity	Runoff peak	Mobilized material	Catchment area (km^2^)	Average fan slope	Buildings damaged	Reported loss (€)
Schnannerbach (Pettneu am Arlberg, Austria)	Fluvial sediment transport	3 m/sec	20 m^3^/sec	35,000 m^3^	6.6	13%	16 (15 used)	480,928
Stubenbach (Pfunds, Austria)	Fluvial sediment transport	Not available	Not available	Not available	29.5	10%	60	11,423,257
Fimberbach (Ischgl, Austria)	Fluvial sediment transport	3,2 m/sec	65 m^3^/sec	3.000 m^3^	66.3	3%	47 (37 used)	11,360,010
Plimabach (Martell, Italy)	Debris flow	Not available	Not available	Not available	Not available	Not available	61 (51 used)	8,500,000

## Results

### The making of the PVI

A preliminary list of indicators (Table 2) was derived based on previous indices (PTVA), expert judgement, literature review and study of damage documentation of past events. Some of the indicators are similar to the ones used in PTVA, however, many additional indicators have been identified that have been considered relevant to the impact of torrential hazards to buildings. The relevance of some of the indicators was additionally confirmed through laboratory experiments^35,36^:

**Table 2:** Summary table of raw data characteristics.

	Code	Data	Type	Source	Uncertainty (low/medium/high)
General information	A	Area	Numeric	GIS buildings shapefile	Low
	H	Height	Numeric	Field survey	Low
	I	Intensity (Height of water and debris)	Numeric	Assessed from damage photos	High
	D	Damage (€)	Numeric	Authorities/municipality	Low
Vulnerability Indicators	WV	Water Vulnerability (No of inundated stories/no of total stories)	Numeric	Calculation from intensity and number of floors	Medium
	MAT	Material	Categorical	Field survey	Low
	FLO	Number of floors	Numeric	Field survey	Low
	RAI	Raised 1^st^ floor	Binary	Field survey	Low
	MAI	Maintenance status	Categorical	Field survey	High
	WALL	Wall thickness	Numeric	Field survey	Medium
	ORI	Orientation	Categorical	Assessed from orthophoto	Low
	GEOM	Geometry	Categorical	Assessed from orthophoto	Low
	PROP	Protruding parts	Binary	Field survey	Low
	AUX	Auxiliary buildings	Binary	Field survey	Low
	LOC_OP	Location of openings	Categorical	Field survey	Low
	H_OP	Height of openings	Numeric	Field survey	Low
	Q_OP	Quality of openings	Categorical	Field survey	High (expert judgment)
	BAS	Existence of basement	Binary	Field survey	Medium (sometimes the surveyor has to assume that there is or there is no basement)
	BAS_OP	Basement openings	Binary	Field survey	Low
	DIS	Distance between buildings	Categorical	Field survey	Medium (it depends on the judgement of the surveyor)
	GR	Steepness of the ground	Categorical	Field survey	Medium (it depends on the judgement of the surveyor)
	SUR_Wh	Height of surrounding wall in relation to the expected debris/water height	Categorical	Field survey	High
	SUR_Wm	Material of surrounding wall	Categorical	Field survey	Medium (different types of walls and combination of materials uncertainty related to how they react to the impact)
	SUR_B	Natural or artificial embankment/protection	Binary	Field survey	Low
	SUR_V	Surrounding vegetation	Categorical	Field survey/Orthophoto	Low
	ROW	Row of buildings towards the flow	Numeric	Assessed from orthophoto	Low
	Ex	Exposure	Numeric	Calculated from intensity	High

In more detail, one of the case studies that are referred to herein (Schannerbach, Pettneu am Arlberg, Austria), was used as a case study. The catchment was replicated in the laboratory in a scale 1:30. Three of the buildings that were damaged during the 2005 event were equipped with sensors to record the impact pressure on each wall. The event of 2005 was simulated in the laboratory and was repeated under 4 scenarios: (i) with buildings surrounding the three buildings under investigation, (ii) without surrounding buildings, (iii) with openings (windows and doors) and (iv) without openings (plain walls). The results clearly showed that the distance from the torrent channel, the existence of surrounding buildings, the height of the debris and the existence of openings may affect the impact pressure on the walls in different ways^35,36^. The scoring of the indicators is described in detail in the method chapter below.

### Selection and weighting of vulnerability indicators

For each case study indicators were collected for the buildings that experienced damage during the considered events. Some of the indicators were collected during field work, some others were available from the authorities and some were interpreted from photographs.

**Figure 2:**
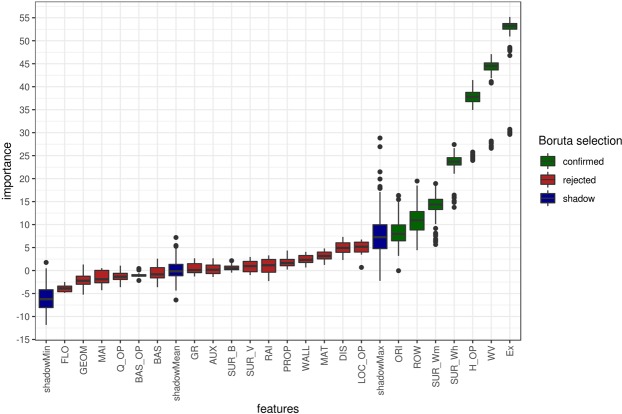
Attribute importance of confirmed and rejected indicators based on Boruta feature selection.

In more detail, indicators were collected for a total of 163 buildings. Details are provided in Table 2. For each of these buildings the degree of loss (the ratio between the reconstruction value of the building and the monetary loss) following specific events was known. Each building was assigned with a score for each indicator as described in detail in the method section. The feature importance of each indicator on the degree of loss for each building was obtained using the random-forest based all-relevant feature selection algorithm Boruta^37^. The Boruta selection revealed two important pieces of information: (a) the relevant indicators that may be used in the making of the PVI and (b) the relative importance of these indicators in the physical vulnerability of the buildings (Fig. 2). According to the Boruta feature selection, a large number (15) of the indicators were rejected whereas only seven indicators perform better than their shuffled duplicates (shadow features) in explaining the degree of loss (Table 3). Consequently, the 15 rejected indicators cannot be used for the calculation of the PVI.

**Table 3:** Indicators to be considered in the index and their weights.

Indicators	Code	Weight
Exposure	Ex	0.28
Vulnerability to water intrusion	WV	0.23
Height of openings	H_OP	0.20
Height of surrounding wall	SUR_Wh	0.12
Material of surrounding wall	SUR_Wm	0.07
Building row towards the torrent	ROW	0.06
Orientation	ORI	0.04

Weights were subsequently calculated for the selected indicators as the relative contribution of each relevant feature to the total importance of all relevant features by dividing the respective features’ median importance by the sum of median importances for all relevant features, resulting in the weightings shown in Table 3.

**Figure 3:**
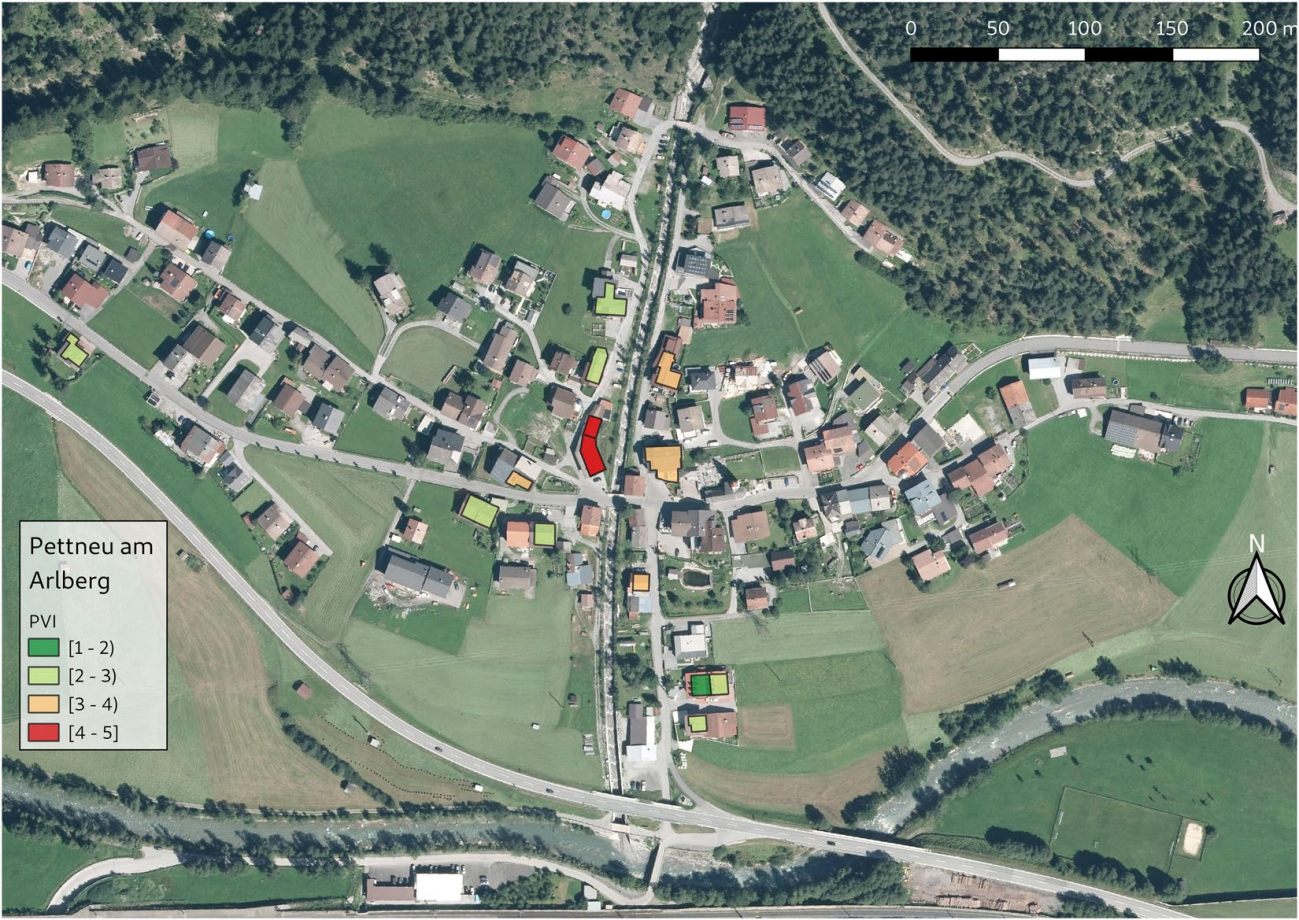
PVI for Schannerbach (municipality of Pettneu am Arlberg, Austria). Source background map: Geodata Web Services [https://gis.tirol.gv.at/arcgis/services/Service_Public/orthofoto/MapServer/WMSServer?request=GetCapabilities&service=WMS], Land Tirol 2019, last access: 04.09.2019, license CC-BY 4.0.

**Figure 4:**
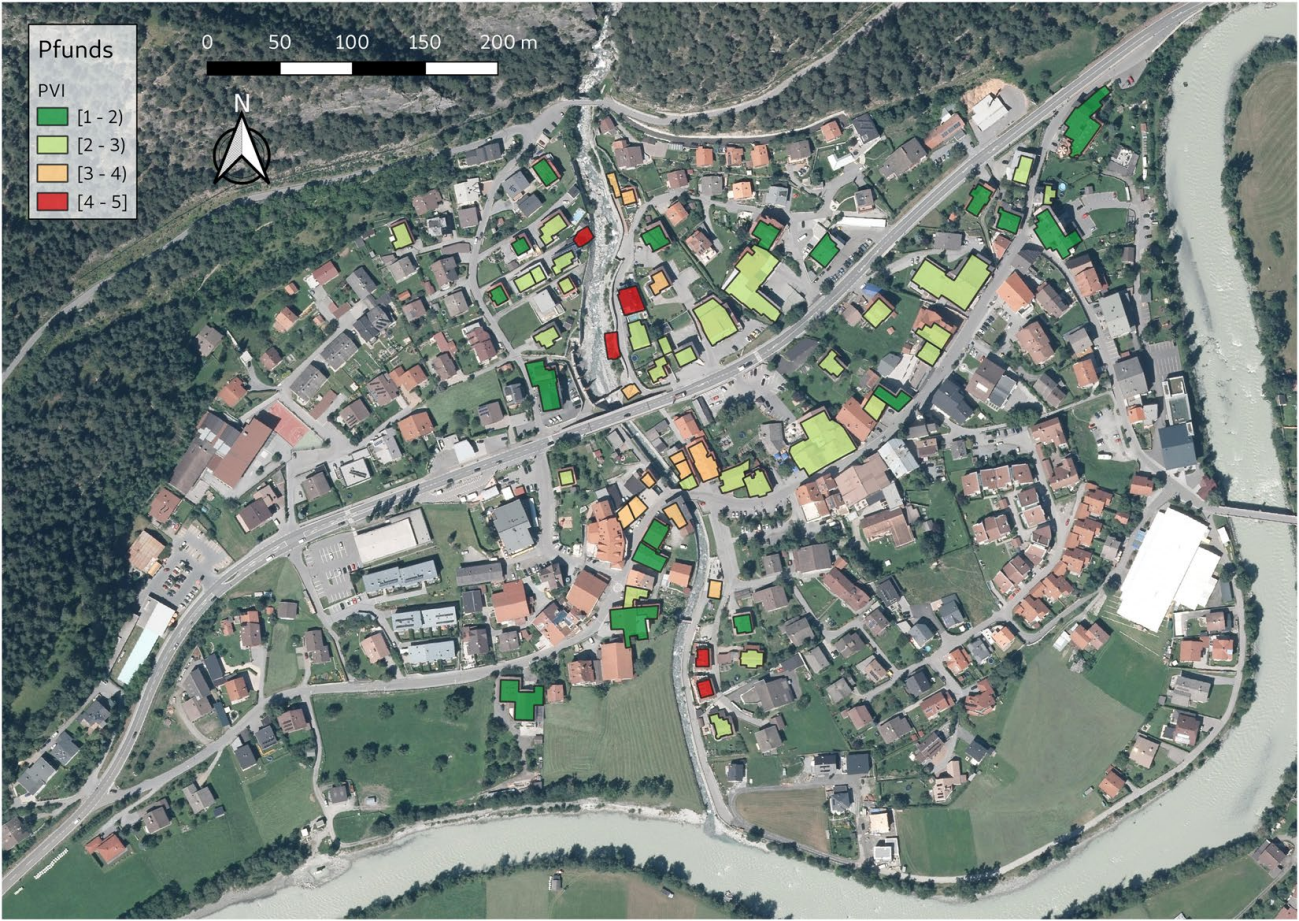
PVI for Stubenbach (municipality of Pfunds, Austria). Source background map: Geodata Web Services [https://gis.tirol.gv.at/arcgis/services/Service_Public/orthofoto/MapServer/WMSServer?request=GetCapabilities&service=WMS], Land Tirol 2019, last access: 04.09.2019, license CC-BY 4.0.

**Figure 5:**
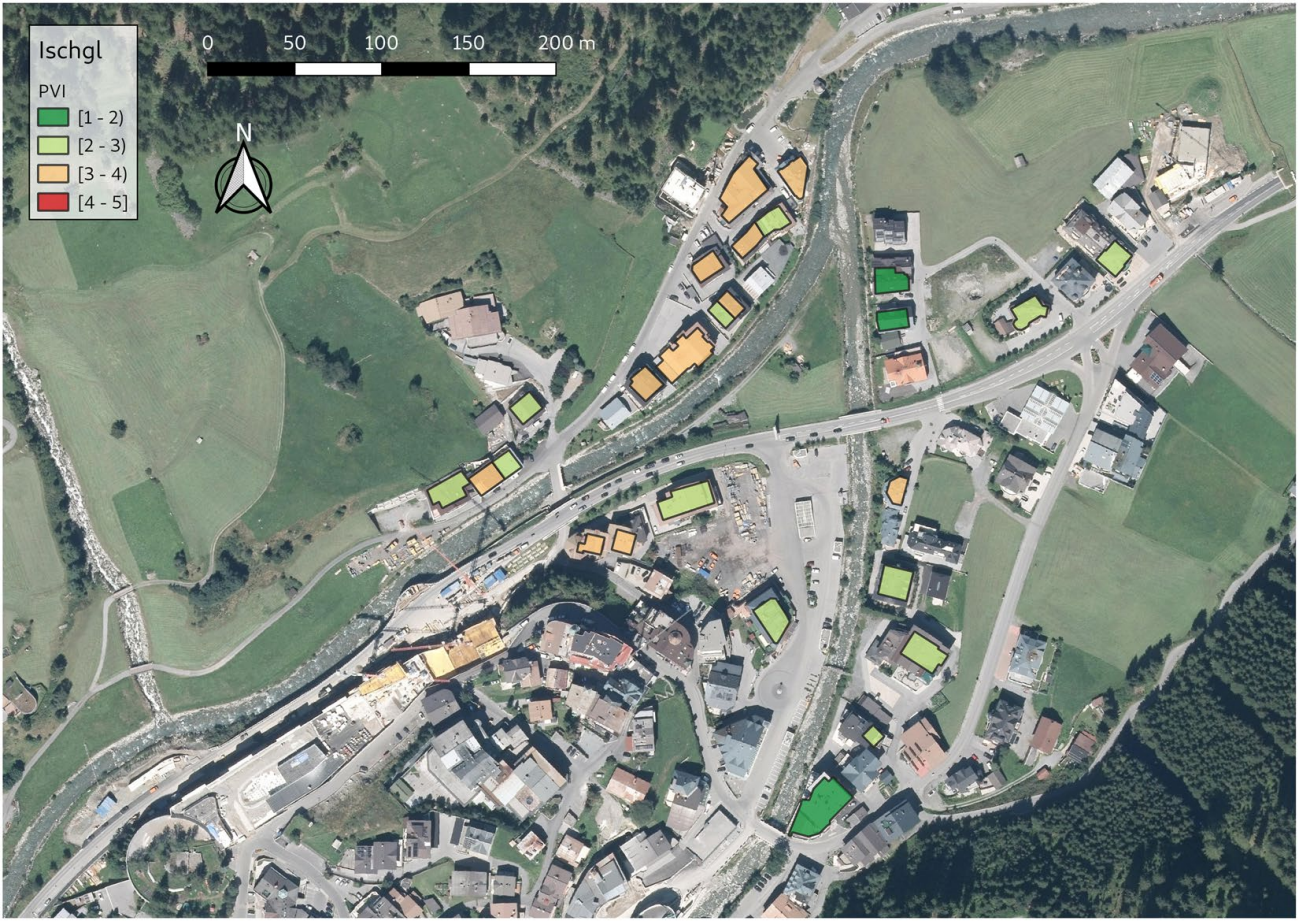
PVI for Fimberbach (municipality of Ischgl, Austria). Source background map: Geodata Web Services [https://gis.tirol.gv.at/arcgis/services/Service_Public/orthofoto/MapServer/WMSServer?request=GetCapabilities&service=WMS], Land Tirol 2019, last access: 04.09.2019, license CC-BY 4.0.

**Figure 6:**
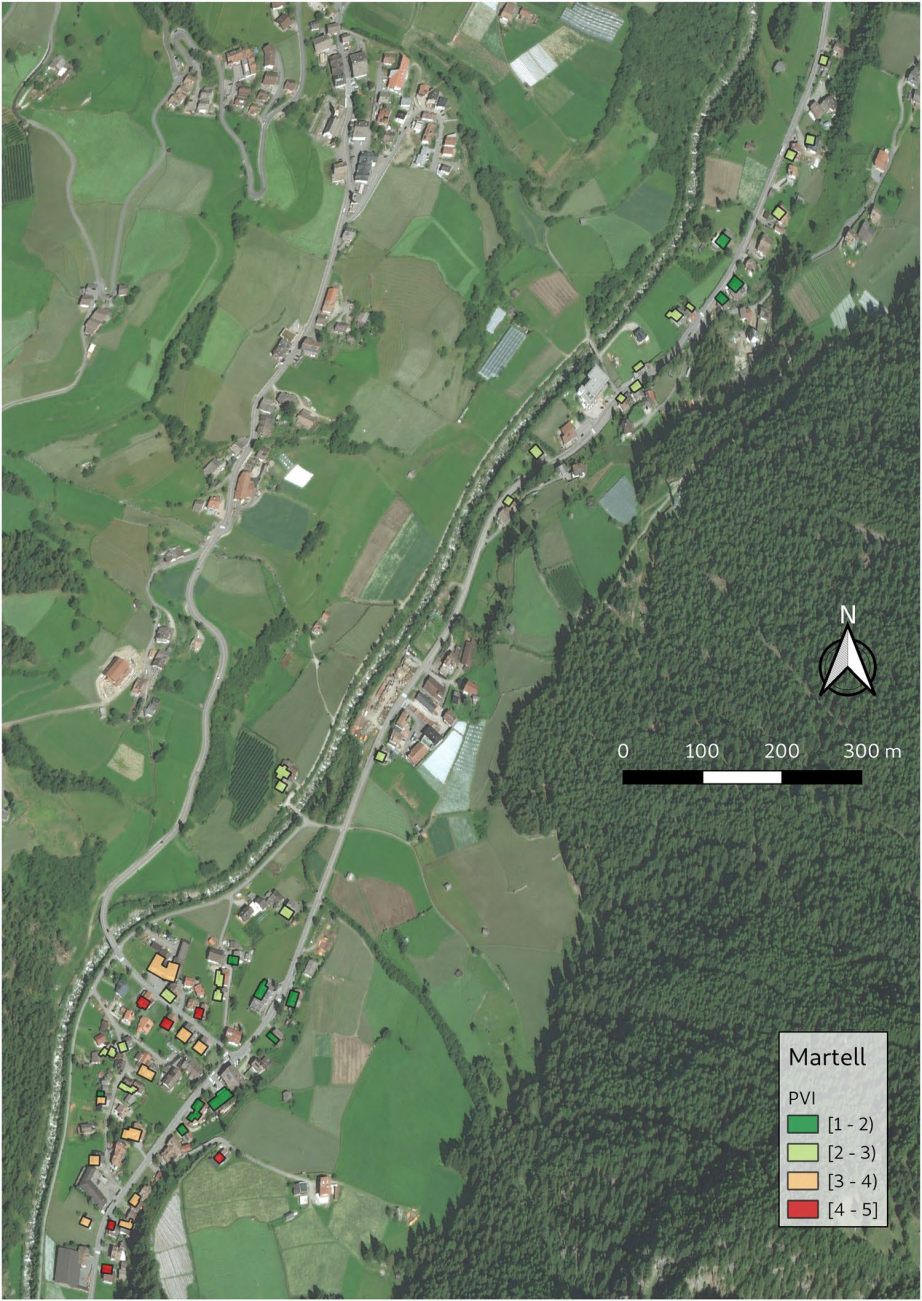
PVI for Plimabach (municipality of Martell, Italy). Source background map: Geodata Web Services [http://geoservices.buergernetz.bz.it/geoserver/ows?service=wms&version=1.3.0&request=GetCapabilities], Autonomous Province Bolzano-South Tyrol 2019, last access: 04.09.2019, license CC-BY 4.0.

Based on these results, the PVI equation has been finalized and is provided in the methods chapter (Eq. 5). Based on this equation, the PVI for each building was calculated in the four case study areas and the spatial pattern of physical vulnerability is shown in Figs 3, 4, 5 and 6. ArcGIS 10.2 and QGIS 3.8 were used for the creation of the maps.

The results of the physical vulnerability assessment can be cartographically displayed by using GIS. A database including all the relevant indicators may have multiple uses and allows regular updating. By visualizing vulnerability assessment results, a question that arises is related to the classification method used. The classification method used in Figs 3–6 for map generation was based on equal intervals, meaning that the difference between the high and the low values of each class is the same. The spatial pattern of the PVI shows that the buildings with very high PVI are located very close to the hazard source, however, buildings with medium or low PVI do not have a specific pattern. A discussion about the available classification methods and an interpretation of the results, as well as an interpretation of the maps and their potential application follows in the discussion chapter.

## Discussion and Conclusions

**Figure 7:**
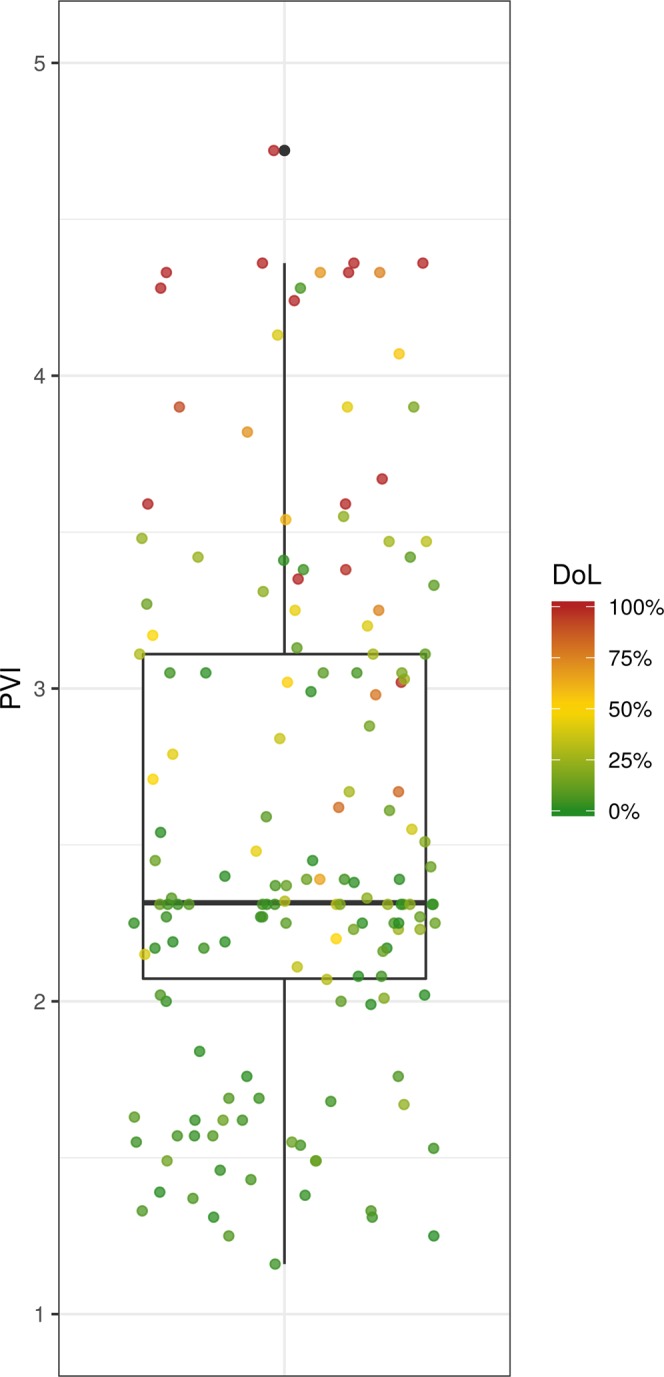
The PVI for each building and the resulting degree of loss in the four case studies from the European Alps.

This paper describes the making of a new Physical Vulnerability Index (PVI) for buildings exposed to dynamic flooding in mountain areas and its application in four case studies in the European Alps. The making of the index required the statistical analysis of the correlation between different building characteristics (vulnerability indicators) and the degree of loss following past disastrous events. As far as the reliability of the index is concerned, in Fig. 7 it is shown that for the four case studies the PVI is in accordance with the real degree of loss. In detail, the PVI is higher for buildings that experienced a high degree of loss and lower for buildings that faced milder losses.

The Boruta feature selection showed which indicators are relevant and should therefore be included in the PVI. Even if some indicators have often been reported as relevant in the literature, they were not able to explain the degree of loss in the specific dataset. Therefore, with a different data set based on buildings that show a higher variation in some characteristics the results would probably be different. For this reason, it is recommended that the non-relevant indicators should be collected in more case study areas and used in the future to validate the present results. It is possible that with a larger dataset the results of the Boruta selection are different and more indicators may be considered relevant.

**Figure 8:**
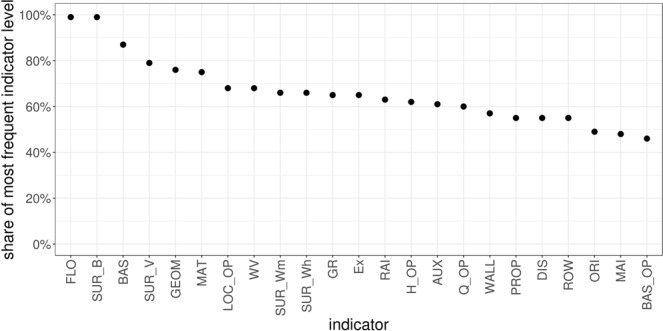
The share of the most frequent factor level for the set of indicators used (for the abbreviation of the indicators see Table 2).

The variation of the different indicators for the present dataset is shown in Fig. 8. It can be concluded that indicators such as the number of floors, the existence of a natural surrounding barrier, and the existence of a basement may have been excluded because in the specific case study most of the buildings had the same scoring for these indicators. Consequently, these indicators do not contain enough discriminatory information. On the other hand, indicators such as the level of the maintenance, the existence of basement openings and the wall thickness were unable to explain the degree of loss although their scoring varies considerably within the dataset. This leads to the conclusion that although the use of indices is seemingly not dependent on empirical data, empirical data are necessary for the weighting of indicators in the first place. Thus, the resulting equation for the calculation of the relative vulnerability index at the moment is still case study specific. However, by including more data from different locations, the weighting will change and a universal, robust index may be developed that can be used in any area where no event record is available. At the local level, however, the index can still show the relative vulnerability of buildings even if indicators such as the existence of basement are not included in the calculation.

The spatial pattern of the new PVI in Figs 3–6 shows the buildings with the highest PVI in red colour. Based on this map building owners can consider the adoption of local adaptation measures such as sealing of basement windows, building of surrounding wall which will change the scoring of the indicators and reduce the vulnerability index. Local adaptation measures are low-cost and efficient measures that can keep water and material outside of the building reducing in this way structural damage, damage of the content and threat to life. It is obvious from the maps that the PVI depends on many factors other than the location of the building. The distribution of PVI is not related only to the distance between the building and the torrent. It is noteworthy that the vast majority of the buildings in the municipalities of Pettneu am Arlberg, Pfunds and Martell are residential (Figs 3, 4 and 6). On the contrary, the vast majority of the buildings in Ischgl (Fig. 5) are hotels with a very high density of population mainly in winter but also in summer months. The overlay of the vulnerability map with information regarding the density of the population or the location of vulnerable groups (children, elderly) could be used by emergency services as a basis for planning their response to a crisis. The vulnerability maps may be used by local emergency services by locating vulnerable groups living in buildings with high PVI without delay following the event. In case of clusters of buildings with high PVI (which is not the case in our maps), local authorities could consider the construction of structural measures to protect this cluster of buildings.

The resulting vulnerability maps and the corresponding decisions for disaster management are very much dependent on the classification method of the vulnerability categories^38^. In Figs 3–6 the “equal interval” classification scheme is used. The “equal interval” method is based on the principle that every class has the same difference between the lower and the higher value. In this way, the presented information is easy to interpret and to be comprehended by a non-technical audience. A different classification scheme, however, such as the “quantile” mode (each class contains an equal number of features) is better for setting priorities, e.g. the top 10% of vulnerable buildings will be retrofitted. The “standard deviation” scheme (classes based on how much a feature’s attribute varies from the mean), on the other hand, may be used in case PVI values over a certain level may be considered unacceptable by the authorities. However, by using the “standard deviation scheme” one must consider that the PVI is an abstract relative value and the average value will be skewed by the very low vulnerability values.

The present paper demonstrated the stepwise development of an index to assess the physical vulnerability of buildings to dynamic flooding (including hyper-concentrated flow, debris flow, etc.). However, it is important to stress that the presented research and results were subject to a number of assumptions and limitations: (a) the data were collected mainly in the field. Limited visibility due to vegetation or difficulty to assign buildings in a specific category is one of the main sources of uncertainty; (b) the degree of loss is the ratio between the monetary damage and the reconstruction value of the building. The degree of loss may seem low if the building for any reason (e.g. many floors) has a very high reconstruction value; (c) the intensity of the process has been often identified from photos that were not always of an excellent quality.

Finally, the main outcomes of the study may be outlined as follows:

- It is possible to transfer methods for vulnerability assessment from one hazard type to another.
- The relevant features (indicators) required for such an assessment are less than it has been initially suggested.
- Indicator-based methods are simple methods that may support decision-making regarding disaster risk reduction in anticipation of change.
- An element at risk database containing indicators regarding the physical vulnerability of buildings to multiple hazards may be a valuable tool in the hands of local authorities, emergency planners, engineers, scientists and insurance companies.
- The method can be transferred to areas with similar architecture without newly adapting the weights even if no record of respective dynamic flooding is available. In the future, using additional data from case studies with different building types and architecture the PVI can become generic and be used anywhere in the world.
  The present paper opens the way for future research including the following:
- The method can be used in order to assess the BBB (Build Back Better) of settlements affected by natural hazards. The PVI index can be assigned to each building using data before and after the event provided that the data are available.
- More research has to be done to enable access to more and better quality data. Innovative methods for data collection as well as post-event documentation are required.
- Data from future events have to be collected continuously to improve the statistical analysis of the indicators and eventually the weighting. Additional data may show that more indicators are relevant and have to be used for the calculation of the PVI. For this reason, it is recommended that detailed sets of indicators including relevant and non-relevant indicators are collected.
- The use of additional resilience indicators has to be considered. Damaged buildings lose their functionality due to a number of characteristics including the type of floor, the location of vital equipment related to the electricity network, the heating or the water supply, etc. This information should be also included in an inventory database of elements at risk.
- The index may be expanded to include vulnerability indicators for more hazard types enabling in this way a multi hazard approach to physical vulnerability.

Finally, the physical vulnerability of buildings should be analysed and assessed in a wider framework of vulnerability and should be seen in an institutional context and in combination with socio-economic data.

## Methods

The construction of the PVI is based on the recommendations of the Handbook on Constructing Composite Indicators^20^.

### Theoretical framework

The theoretical framework should define the concept, the indicators and the selection criteria^20^. Herein, the theoretical framework should include a definition of vulnerability. The UNISDR^39^ definition of vulnerability is used and the method is adjusted to it. Thus, vulnerability comprises “the conditions determined by physical, social, economic and environmental factors or processes which increase the susceptibility of an individual, a community, assets or systems to the impacts of hazards”.

### Selection of variables

The approach presented here is based on the PTVA, a method for vulnerability assessment originally developed for tsunami hazards. Given the differences between the two processes (tsunami and torrential hazards) the collection of additional indicators is considered essential. The indicators considered are shown in Table 2.

### Scoring and normalization of data

Categorical, numeric and binary raw data were collected. Each indicator was given a score which allows the comparison with the other indicators (normalization). The scores are based on the PTVA method^34^. The Relative Vulnerability Index (RVI) developed in PTVA^34^ is given by the following equation:

1 $$RVI=1/3\,WV+2/3\,SV$$

where *WV* is the vulnerability to water intrusion (number of inundated levels/number of all levels), and *SV* is the Structural Vulnerability which is the product of Building Vulnerability *(BV)*, Protection *(Prot)* and Exposure *(Ex)*:

2 $$SV=BV\times Ex\times {\Pr }ot$$

Dall’Osso *et al*.^34^ assume that the overall Relative Vulnerability Index (RVI) is equal to 2/3 of the SV and 1/3 of the *WV*. We consider this assumption arbitrary. Therefore, we use *WV* and *Ex* as any other indicators and we test their relevance using statistical methods.

Each indicator related to the *Bv* (Table 4) and to *Prot* (Table 5) was given a score ranging from 1 to 5 according to whether and to which degree the specific characteristic contributes to a decrease or an increase of the overall physical vulnerability of the building respectively. The *Ex* is given by the depth of water expected at the building location. The scoring of the indicators related to the *WV* and the *Ex* are shown in Table 6.

**Table 4:** The scoring of indicators related to the Building Vulnerability (*Bv*).

Indicator	1	2	3	4	5
MAT	Stone		Mixed	Brick	Wood
MAI	Very good (new)	Good		Average	Very bad
FLO	Multi-storey building				Single-storey building
RAI	Yes				No
WALL	1 m		0.5 m		0.3 m
ORI	Parallel to the flow		Square building		Perpendicular to the flow
GEOM	Solid Long parallel to the flow			Long Perpendicular to the flow	Complicated
PROP	No				Yes
AUX	No				Yes
LOC_OP	Back side or no openings		Side		Flow side
H_OP	More than or equal to the expected intensity: H > I or H = I				Less than the expected intensity: H < I
Q_OP	Good/very good		Average		Bad (or all glass facade)
BAS	No				Yes
BAS_OP	No, or covered			Yes, no flow side	Yes, flow side
DIS	Attached		Large	Medium	Small
GR	Flat		Mild		Steep

**Table 5:** The scoring of indicators related to the protection (*Prot)*.

Indicator	1	2	3	4	5
Surrounding vegetation (SUR_V)	In forest	High bushes/dense vegetation	Single trees	Low vegetation	No vegetation
Building row towards the torrent (ROW)	>5	4	3	2	1
Natural or artificial barrier (e.g. embankment)SUR_B	Yes				No
Material of surrounding wall (SUR_Wm)	Stone/brick/concrete	Mixed (concrete + wood)	Wooden fence	Wire fence	No wall
Height of surrounding wall (SUR_Wh)	H > 80% of water depth	60% < H < 80% of water depth	40% < H < 60% of water depth	20% < H < 40% of water depth	0% < H < 20% of water depth

**Table 6:** The scoring of indicators related to the vulnerability to water intrusion (*WV)* and Exposure (*Ex)*.

Indicator	1	2	3	4	5
Vulnerability to Water Intrusion (WV)	[0, 0.2)	[0.2, 0.4)	[0.4, 0.6)	[0.6, 0.8)	[0.8, 1]
Exposure (Ex)	0–1 m	1–2 m	2–3 m	3–4 m	>4 m

### Weighting and aggregation

The different versions of the PTVA used expert judgement, AHP and questionnaires to define the weight of the indicators. Herein, we perform a different weighting based on the feature importance of each indicator with the actual degree of loss during past events (Table 3).

Determining weights for available input variables is one of the most important aspects of calculating a vulnerability index due to potentially large effects of different feature weights on the outcome. From a statistical point of view this is closely related to the aspect of feature selection in model construction. Given data sparsity in a high-dimensional feature space, removing redundant or irrelevant variables is an important step towards reducing input data dimension and consequently increasing model performance. Essentially, variable selection is characterized by a trade-off between bias and variance: with increasing model complexity, bias decreases while variance increases, eventually leading to overfitting and poor model generalization performance^40^.

Classical machine learning approaches are often targeted towards obtaining the best predictive models given available training data. Therefore, their focus is on finding a minimal number of features that is optimal for classification, thereby solving the so-called *minimal-optimal* problem. While this may result in best possible predictive performance, any model constructed using a minimal-optimal strategy is merely a black box. This poses some restrictions on interpretability, because minimal-optimal attributes are typically determined by technical factors (i.e. a favourable signal-to-noise ratio) rather than causal relationships between independent features and the dependent variable. However, if the focus is on inference instead of prediction, one is usually primarily interested in understanding the underlying mechanisms linking predictors to the subject of interest, thus, requiring information on all attributes that are somewhat causally related to the target variable. This emphasis on causal significance between the selected feature set and the target variable leads to the *all-relevant* problem. An excellent description and discussion of these two concepts is provided by Nilsson *et al*.^41^.

In the present case, we are interested in identifying all attributes that are – at least in some circumstances – relevant for determining the degree of loss. Therefore, our feature set is obtained by employing Boruta, an all-relevant feature selection algorithm based on random forests^37^. Essentially, Boruta is a permutation-based testing strategy that relies on comparing the relevance of each single feature to randomly shuffled copies of all variables (“shadow attributes”). By conducting two-sided tests of equality on the *Z* score (i.e. the standardized and normalized accuracy loss) between each attribute and the best performing shadow attribute, all features are iteratively flagged as important (i.e. significantly more important than the shadow attributes) or unimportant (i.e. significantly less important than the shadow attributes).

In order to obtain optimal results, random forest hyperparameter tuning was performed prior to using Boruta to define (i) the number of variables to possibly split at in each node, (ii) the minimum node size as well as (iii) the number of trees, using an exhaustive grid search with cross-validation. The resulting Boruta feature selection revealed seven relevant features (Fig. 2).

The equation used for the calculation of the relative vulnerability index used in the present paper is the following:

3 $$PVI={w}_{1}\times WV+{w}_{2}\times Ex+{\sum }_{b=1}^{m}({w}_{b+2}{I}_{b}^{BV})+{\sum }_{p=1}^{n}({w}_{p+m+2}{I}_{p}^{Prot})$$

where *PVI* indicates the Physical Vulnerability Index, *WV* is the vulnerability to water intrusion, *Ex* is the exposure, *I* denotes the indicators used for building vulnerability (superscript *BV*) and protection (superscript *Prot*), *m* and *n* denote the number of relevant indicators for *BV* and *Prot* respectively, and *w* is the weight of each indicator. Weightings (*w*_*i*_) are calculated as relative contribution of each relevant feature to the total importance of all *j* relevant features by dividing the respective features’ median importance (${\tilde{f}}_{i}$) by the sum of median importances for all relevant features:

4 $${w}_{i}=\frac{{\tilde{f}}_{i}}{{\sum }_{j=1}^{N}\,{\tilde{f}}_{i}}$$

Based on the resulting weighting (Table 3) the final equation used for each building is:

5 $${\rm{PVI}}=(WV\times 0.23)+(Ex\times 0.28)+({{\rm{H}}}_{{\rm{OP}}}\times 0.20)+({{\rm{SUR}}}_{{\rm{Wh}}}\times 0.12)+({{\rm{SUR}}}_{{\rm{Wm}}}\times 0.07)+({\rm{ROW}}\times 0.06)+({\rm{ORI}}\times 0.04)$$

where *WV*: the vulnerability to water intrusion, *Ex*: the exposure, H_OP_: the height of windows, SUR_Wh_: the height of the surrounding walls, SUR_Wm_: the material of the surrounding walls, ROW: the building row towards the torrent and ORI: the orientation of the building.

### The PVI index and classification

The choice of the classification method is closely related to the aim of the vulnerability assessment (e.g. priority settings, reinforcement priority or funding allocation, support for decision making) and it can be misleading. The most commonly used classification methods in GIS are natural breaks (Jenks), equal interval, quantile (equal counts) and standard deviation. The choice of the classification method is related to the use of the vulnerability map.

### Presentation and dissemination

In general, indicators and indices may be visualized in tables, bar or line charts or trend diagrams^20^. However, due to the geographical nature of the PVI, and the need to communicate the information to a large number of stakeholders (e.g. local authorities, emergency services and the public) the use of geographical information systems is the most appropriate way of representation.

## Data Availability

The dataset generated and analysed in the present study may become available upon request.
